# Large-Scale Continuous Monitoring of Greenhouse Gases with Adaptive LoRaWAN in CN470–510 MHz Band

**DOI:** 10.3390/s25175349

**Published:** 2025-08-29

**Authors:** Xueying Jin, David Chieng, Pushpendu Kar, Chiew Foong Kwong, Yeqin Li, Yin Wang

**Affiliations:** 1 Next-Generation Internet of Everything Laboratory, Department of Electrical and Electronic Engineering, University of Nottingham Ningbo China, Ningbo 315100, Chinachiew-foong.kwong@nottingham.edu.cn (C.F.K.); yeqin.li@nottingham.edu.cn (Y.L.); 2 School of Computer Science, University of Nottingham Ningbo China, Ningbo 315100, China; pushpendu.kar@nottingham.edu.cn; 3 HealthyPhoton Technology Co., Ltd., Ningbo 315042, China; yin.wang@healthyphoton.com

**Keywords:** sensor network, LoRaWAN, adaptive data rate, GHG monitoring, long-range wireless area network

## Abstract

Continuous and near-real-time monitoring of greenhouse gases (GHGs) is critical for achieving Net Zero emissions, ensuring early detection, compliance, accountability, and adaptive management. To this end, there is an increasing need to monitor GHGs at higher temporal resolutions, greater spatial resolutions, and larger coverage scales. However, spatial resolution and coverage remain significant challenges due to limited sensor network coverage and power sources for sensor nodes, even in urban areas. LoRaWAN, a cost-effective solution that provides long-range and high-penetration wireless connectivity with a low energy consumption, is an ideal choice for this application. Despite its promise, LoRaWAN faces several challenges, including a low data rate, low packet transmission rate, and low packet delivery success ratio, especially when the node density or environment variability is high. This paper presents a simulation-based analysis of a large-scale urban LoRaWAN sensor network operating in the CN470–510 MHz band, which is the only frequency band officially designated for low-power wide-area (LPWA) technologies such as LoRaWAN in China. This study investigates how the node density, sensor measurement update rate (i.e., update interval), and sensor measurement payload size affect two primary performance metrics: the sensor update delivery ratio (DR) and the radio frequency (RF) energy consumption (RFEC) per successful update. The performances of several enhanced adaptive data transmission algorithms in comparison to the conventional ADR+ algorithms are also analysed. The results indicate that both DR and RFEC are significantly influenced by the node density, sensor update rate, and payload size, with the effects being particularly significant under high-node-density and high-update-rate conditions. The analysis further reveals that the ADR-NODE-KAMA algorithm consistently achieves the best performance across most scenarios, providing up to a 2% improvement in DR and a reduction of 10–15 mJ in RFEC per successful sensor measurement update. Additionally, the sensor measurement payload size is shown to have a substantial impact on network performance, with each added sensor measurement contributing to a DR reduction of up to 2.24% and an increase in RFEC of approximately 80 mJ. LoRaWAN network operators can gain practical insights from these findings to optimize the performance and efficiency of large-scale GHG monitoring deployments.

## 1. Introduction

The widespread use of fossil fuels, large-scale deforestation, and various land-use changes have resulted in a continuous rise in atmospheric greenhouse gas (GHG) levels, thereby triggering global climate change [1]. In 2023, the global average surface concentration of carbon dioxide (CO_2_) was 420.0 parts per million (ppm), that of methane was 1934 parts per billion (ppb), and the concentration of nitrous oxide was 336.9 parts per billion, which represent increases of 51%, 165%, and 25%, respectively, compared to pre-industrial values. Furthermore, 2023 marked the 12th consecutive year with an annual carbon dioxide growth exceeding 2 ppm [2]. To reduce GHGs, China has set a goal to achieve “peak carbon” emissions by 2030 and become “carbon neutral” by 2060 [3].

Continuous and near-real-time monitoring of greenhouse gases is crucial for achieving Net Zero emissions, ensuring early detection, compliance, accountability, and adaptive management. More specifically, GHG monitoring is vital for investigating the global carbon cycle, evolution trends, regional sources and transport, ecological flux estimates, urban or industrial emissions estimates, validations of chemical transport models (CTMs), and emission inventories [4]. Given China’s rapid urbanization, with over 60% of its population now living in cities, it is no surprise that urban areas are major sources of carbon emissions [5] due to energy consumption, transportation, and industrial activities [6]. Effective GHG monitoring can also help cities adopt sustainable practices such as energy-efficient buildings, green transportation, and renewable energy to balance economic growth with environmental protection. By integrating GHG monitoring with smart city technologies (e.g., IoT sensors, AI, and big data), resource use can be optimized, and emissions can be reduced.

A recent APEC report [7] identifies critical gaps in current monitoring strategies, including insufficient sensor coverage across vast or difficult-to-access regions, which limits the ability to capture spatially representative emissions data at fine scales. The report also stresses that IoT technologies are essential for environmental sensing, as they enable automated, resilient monitoring across large networks, ensuring accurate data collection and timely emission control, even in harsh conditions. Consequently, there is a growing demand for cost-effective, scalable, and reliable solutions for GHG measurement and monitoring that can operate across diverse geographic and environmental conditions. The authors of [4] further emphasize that effective monitoring may require the following scales and resolutions: 1. A temporal resolution, in terms of the sensor measurement update rate, ranging from typical intervals of several hours down to minutes or seconds. 2. A spatial resolution, in terms of distance between sensor nodes, ranging from several kilometres down to a few metres. 3. A coverage scale ranging from local, regional, large, to global. These requirements are particularly challenging due to limited sensor network coverage and power sources over large geographical areas, even in urban environments. Providing such services is generally not commercially viable and often requires support from non-profit initiatives. In addition, achieving a high temporal resolution is technologically challenging.

In this context, low-power wide-area networking (LPWAN) technologies such as LoRaWAN [8] offer a promising solution to the challenges of large-scale, long-term GHG monitoring. Unlike other LPWAN technologies such as NB-IoT, Sigfox, and LTE-M, which operate in the licenced spectrum and are typically controlled by major network operators like China Mobile or China Telecom, LoRaWAN operates in the unlicensed spectrum [9]. This allows independent stakeholders to deploy and manage networks with greater flexibility and at a lower cost. Its ability to provide long-range, high-penetration wireless connectivity with minimal energy consumption makes it well-suited for powering distributed sensor networks. Moreover, the low power requirements facilitate integration with renewable energy sources, further enhancing the sustainability and scalability of LoRaWAN for environmental sensing applications.

Despite these promising features, LoRaWAN faces several challenges, such as a low data rate, low packet transmission rate, and low packet delivery ratio, especially when the node density or environmental variability is high. A new investigation into the feasibility of this technology for continuous GHG monitoring, as well as into techniques that can be adopted to improve performance, is therefore required.

In this paper, the modelling and performance analysis of a large-scale LoRaWAN network in the context of continuous GHG monitoring in China’s CN470–510 MHz band are presented. It investigates how the node density, sensor update rate, and sensor measurement payload size affect the network’s sensor measurement update delivery ratio (DR) and RF energy consumption (RFEC). The paper also presents the effects of several enhanced adaptive data transmission algorithms on DR and RFEC. The contributions of this work are summarized as follows:

- The key requirements for effective continuous GHG monitoring are outlined, and the feasibility of LoRaWAN technology meeting these requirements is evaluated.
- Specifically, this paper conducts an in-depth theoretical analysis of sensor measurement uplink capacity by examining the constraints imposed by the LoRaWAN protocol, including limitations on the maximum payload size, time on air (ToA), and regulatory duty cycle restrictions.
- A comprehensive simulation model is presented to investigate the effects of node density, sensor update rate (or interval), and sensor payload sizes on the network’s overall sensor update DR and RFEC per successful sensor update in China’s CN470–510 MHz band.
- Several statistical enhancements of the conventional uplink adaptive data rate algorithm are proposed.
- The results indicate that both DR and RFEC are significantly influenced by the node density, sensor update rate, and payload size, with the effects being particularly noticeable under high-node-density and high-update-rate conditions. The analysis further reveals that the ADR-NODE-KAMA algorithm consistently achieves the best performance across most operating scenarios, providing up to a 2% improvement in DR and a reduction of 10–15 mJ in RFEC per successful sensor update. Additionally, the sensor measurement payload size is shown to have a substantial impact on network performance, with each added sensor measurement contributing to a DR reduction of up to 2.24% and an increase in RFEC of approximately 80 mJ.

LoRaWAN network operators can gain practical insights from these findings to optimize the performance and efficiency of large-scale GHG monitoring deployments. Operators can use this knowledge to fine-tune deployment strategies, such as deciding spatial and temporal resolutions and payload configurations, to reduce congestion and energy waste. The superiority of the ADR-NODE-KAMA algorithm provides a means for improving network reliability and energy efficiency. Additionally, understanding the performance trade-offs associated with adding sensor measurements helps operators make informed decisions about system scalability and sensing resolution, particularly in the context of regulatory constraints such as the 1% duty cycle in the CN470–510 MHz band.

## 2. Related Works

The application of LoRaWAN for large-scale monitoring systems has been extensively studied in recent years, with research focusing on energy efficiency, network optimization, and performance evaluation. Slabicki et al. [10] developed an open-source framework called FLoRa for simulating LoRa networks in OMNeT++ and evaluated the adaptive data rate (ADR) mechanism for dense IoT deployments. They proposed an improved ADR algorithm to enhance network performance under variable channel conditions, demonstrating significant improvements in reliability and energy efficiency. The framework and ADR+ are adopted in this work as one of the benchmark algorithms.

Gava et al. [11] proposed a resource optimization methodology for LoRaWANs, using variable neighbourhood search (VNS) and minimum-cost spanning tree algorithms to reduce implementation and maintenance costs. Their study optimized parameters such as the spreading factor (SF), bandwidth, and transmission power to minimize energy consumption and data collection time. The results demonstrated that optimizing these parameters can significantly enhance network performance, especially in scenarios with varying node densities.

Sherazi et al. [12] investigated the energy efficiency of LoRaWAN in industrial environments, focusing on battery life, replacement costs, and the potential of energy harvesting to extend device lifetimes and reduce operational costs. Their study highlighted the trade-offs between sensing intervals, battery life, and damage penalties, demonstrating the potential for significant cost savings through optimized energy management.

Griva et al. [13] assessed the performance of LoRa-based IoT networks in rural and urban scenarios, using different path loss models to simulate various deployment environments. Their study highlighted the impact of key parameters such as the transmission power, spreading factor, and gateway placement on network performance metrics like the data extraction rate (DER) and network energy consumption (NEC). The findings indicated that optimizing these parameters is crucial for efficient network deployment in diverse environments.

Fang et al. [14] conducted in situ measurements of atmospheric CO_2_ at four WMO/GAW stations in China using cavity ring-down spectroscopy instruments. They analysed the diurnal, seasonal, and interannual variations in CO_2_ mole fractions, providing insights into the regional carbon cycles and underlying fluxes. Their work emphasized the importance of understanding local sources and sinks for accurate atmospheric CO_2_ monitoring, which is crucial for developing reliable GHG monitoring systems.

Eich et al. [15] presented a proof-of-concept study for environmental monitoring using LoRaWAN, focusing on temperature, humidity, and CO_2_ concentration measurements in greenhouse environments. Their work evaluated the reliability of LoRaWAN communication in real-world agricultural settings and highlighted both the challenges and best practices associated with long-term deployment. While the authors focused on proof-of-concept deployments in controlled greenhouse environments, this work extends their investigation by modelling a large-scale urban LoRaWAN network. It emphasizes continuous, near-real-time GHG monitoring and analyses the performance impact of network parameters, such as the node density, sensor update rate, and payload size, within China’s CN470–510 MHz band.

These studies collectively provide a comprehensive understanding of the potential and challenges of deploying LoRaWAN for large-scale monitoring applications. They emphasize the importance of adaptive and optimized network configurations to achieve energy efficiency, extended device lifetimes, and reliable communication, which are essential for near-real-time large-scale GHG monitoring systems. This work extends the above studies by modelling deployment scenarios based on the China CN470 specification [11] in the context of continuous GHG monitoring. Several enhancements to uplink adaptive data algorithms with a uniform sensor update rate are proposed, and their effectiveness is compared in terms of DR and RFEC.

## 3. Methodology

This section presents the methodology, theoretical framework, and performance metrices used in this study.

### 3.1. FLoRa

Framework of LoRa (FLoRa) is a comprehensive simulation framework designed to conduct end-to-end simulations of LoRa networks comprising LoRa nodes, gateways, and network servers [16] It leverages the capabilities of the OMNeT++ network simulator and integrates components from the INET framework. The LoRaWAN nodes can send packets to gateways via multiple channels simultaneously, according to LoRaWAN specifications [17]. Both the network server and the nodes are capable of dynamically adjusting configuration parameters via ADR mechanisms. With this capability, especially at the network server, network operators can design their own ADR algorithms according to their operating requirements. In addition, each node is capable of gathering statistics on energy consumption. Although Flora supports multiple gateways, this study is limited to one gateway to simplify the analysis on the impact of node densities, the sensor update rate, and the sensor payload size.

Several simulation tools have been developed for studying LoRa networks, each with distinct strengths and limitations. LoRaSim, a widely used Python-based discrete-event simulator, offers scalability and collision analysis on a 2D grid; however, its simplified physical-layer model and lack of ADR mechanisms limit its realism and energy modelling capabilities. The NS-3 Network Simulator (NS-3) LoRaWAN module offers a comprehensive network simulator with configurable PHY/MAC layers, ADR support, and multi-device class capabilities. However, it demands significant effort to implement detailed energy consumption models and is complex to use. LoRaWANSim, which is implemented in MATLAB, provides a detailed physical-layer model for LoRaWAN, but it is constrained to single-channel, Class A devices and lacks support for multi-channel operations or advanced ADR strategies. For the reasons outlined above, the FLoRa model developed by Slabicki et al. [10] has since become one of the foundational works cited by researchers for extending or benchmarking LoRaWAN simulations.

### 3.2. LoRa Physical Layer

In this work, only the CN region parameters are considered. The CN470–510 MHz band serves as the primary band in China, which comprises 96 uplink channels and 48 downlink channels. In some regions, the CN779-787 MHz band may also be utilized as an additional band. Both bands are capable of supporting a 125 kHz bandwidth. When it comes to the coding rate, both 4/5 and 4/8 are supported, where 4/8 is recommended for noisy environments. Although according to [17], the transmit power should be limited to 14 dBM, for some industrial applications, the limit can go up to 27 dBm (500 mW) with China’s Ministry of Industry and Information Technology (MIIT)’s approval. Table 1 describes the physical layer parameters adopted in this study, which are in accordance with LoRaWAN Alliance [17]. In this work, only uplink parameters are considered, because the primary objective is to evaluate the performance of sensor measurement updates in a continuous GHG monitoring scenario. In such deployments, uplink traffic, which transmits measurement data from distributed sensor nodes to a centralized server, constitutes the dominant share of communication. In contrast, downlink transmissions, such as acknowledgements or configuration commands, are typically infrequent and event-driven, particularly in Class A LoRaWAN devices. This methodology is in line with prior studies, where key performance metrics such as DR and RFEC are primarily derived from uplink communication [10,11,13].

### 3.3. Path Loss Modelling

According to [19] the received power $P_{rx}$ is computed by

(1) $$P_{rx}=P_{tx}+GL-PL$$

where $P_{rx}$ is the received power, $P_{tx}$ is the transmit power (TP), GL includes all gains and losses, and PL represents the path loss. In addition, the log-distance path loss model with shadowing is commonly used to model the loss of signal strength during propagation. The path loss over a certain distance, $PL\left(d\right)$, is represented by

(2) $$PL\left(d\right)=\bar{PL}\left(d_{0}\right)+10nlog\left(\frac{d}{d_{0}}\right)+X_{\sigma }$$

where $\bar{PL}\left(d_{0}\right)$ represents the mean path loss measured in dB, n is the path loss exponent, and $X_{\sigma }$ denotes zero-mean Gaussian distribution loss with the standard deviation σ.

In this paper, since we focus on the urban scenario, the typical values of the propagation exponent, n, and shadow fading, σ, are shown in Table 2.

$\bar{PL}\left(d_{0}\right)$ is the path loss at $d_{0}$, calculated using the Hata urban model at 490 MHz [20], which is given by

(3) $${PL}_{urban}=69.55+26.16\log_{10}f_{c}-13.82\log_{10}h_{gw}-a\left(h_{node}\right)+(44.9-6.55\log_{10}h_{gw})\log_{10}d$$

where $f_{c}$is the carrier frequency (this model is valid for 150 MHz ≤ f ≤ 1500 MHz).

Small-scale fading effects such as multipath, Doppler shift, and mobility-induced variations are not explicitly modelled in this study, as all sensor nodes are assumed to be stationary and are placed in elevated, line-of-sight or near-line-of-sight conditions, which is typical in fixed environmental sensing deployments. While this assumption limits the model’s ability to capture fine-grained physical layer variations, it provides a reasonable trade-off between realism and scalability of the model.

### 3.4. Sensor Measurement Update Rate per LoRaWAN Node

The sensor measurement update rate per LoRaWAN node initially depends on the packet transmission rate, which is defined as the number of packets that can be transmitted from the sensor node to the gateway within a period of time. Equation (4) depicts the required period for a sensor node to transmit one message, $T_{packet}$, which is also referred as time on air (ToA) according to [18].

(4) $$ToA\;or\;T_{packet}=T_{preamble}+T_{payload}$$

(5) $$T_{preamble}=\frac{{(N}_{preamble}+4.25)*2^{SF}}{BW}$$

(6) $$T_{payload}=\frac{N_{payload}*2^{SF}}{BW}$$

where $N_{preamble}$ is the number of preamble symbols, which is equal to 8; 4.25 is the sync word, with 2 symbols and 2.25 additional overhead symbols; $N_{payload}$ is the number of payload symbols; SF is the spreading factor (7 to 12); and BW is the channel bandwidth of 125 kHz [18]. Additionally, $N_{payload}$ is derived as follows:

(7) $$n_{payload}=8+\max\left(ceil\left[\frac{\left(8B_{payload}-4SF+28+16CRC-20IH\right)}{4\left(SF-2DE\right)}\right]\left(CR+4\right),0\right)$$

where $B_{payload}$ is the number of payload bytes, IH = 0 when header is enabled, IH = 1 means no header, and CR represents the coding, where $\frac{2^{SF}}{BW}{=T}_{s}$, i.e., the symbol time.

When $T_{s}$ exceeds 16 ms, the Low Data Rate Optimization(DE) flag is to be set to 1; otherwise, DE = 0 [21]. This low-data-rate optimization is required to mitigate issues like drift during long transmission. Hence assuming a payload size of 59 bytes with CRC enabled (i.e., CRC = 1), the ToA for each spreading factor (SF) is derived as presented in Table 3.

According to [22], the minimum time, $T_{wait}$, defined as the required silent period following a transmission, can be expressed as follows:

(8) $$T_{wait}=\left(\frac{100}{Duty\;Cycle}-1\right)ToA$$

Based on this equation and duty cycle = 1 to comply with the 1% duty cycle regulation in the CN470 band, the computed $T_{wait}$ values for each spreading factor (SF) are presented in Table 4.

Equation (8) highlights the direct relationship between duty cycle constraints and the time on air (ToA), demonstrating how this behaviour is captured in the model.

A successful sensor update also depends on the presence or absence of interference. Transmissions on orthogonal channels, such as those using different spreading factors, are assumed not to interfere. However, collisions occur when non-orthogonal transmissions overlap in time. In FloRa, the capture effect is considered, whereby the stronger of two colliding signals can be successfully decoded if its power exceeds that of the weaker signal by more than 6 dBm and at least five preamble symbols are detected [10]. A MAC-layer backoff mechanism such as CSMA/CA or exponential backoff is not implemented in FloRa. Instead, it follows the LoRaWAN Class A communication model, which employs a pure ALOHA protocol. In ALOHA, nodes transmit immediately upon data availability, and collisions are resolved through the capture effect described earlier. In other words, the channel access is indirectly regulated by duty cycle constraints and transmission intervals.

#### 3.4.1. LoRaWAN Packet Structure

**Figure 1:**
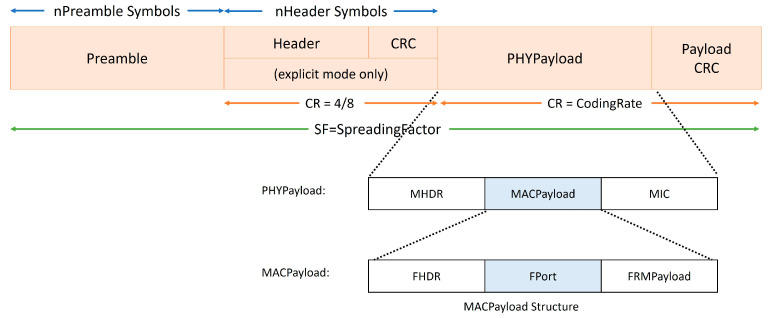
LoRaWAN packet structure [18].

The LoRaWAN packet is illustrated in Figure 1 below.

There are two modes of operation, namely (1) explicit and (2) implicit modes, where the later omits the packet header specifying its structure, such as the payload size and coding rate. The implicit mode is typically used when the packet sizes are uniform. Table 5 depicts a typical uplink LoRaWAN packet structure with its fields’ sizes [23]. The cells coloured in light grey sit in the PHY payload.

As shown, the frame payload is the actual field that carries application-level data such as sensor measurements. According to [17], the maximum MAC payload size is determined by the data rate, as shown in Table 6. Note that N is without an 8-byte optional-frame control field.

When monitoring GHGs across a large area, regardless of the data rate or SF, a fixed payload with a uniform update rate is required in order to maintain a consistent sensor status update, regardless of where the sensor node is deployed. Based on the analysis above, a size of 59 bytes is set as the maximum possible payload size in this study.

#### 3.4.2. Sensor Measurement and Payload Size

In this section, the detection unit, encoding type, and size of various sensor measurement data are analysed in order to understand LoRaWAN’s capability in delivering sensor measurements. Table 7 presents the standard detection units, encoding formats, and data sizes commonly used for greenhouse gases (GHGs) and associated environmental variables. GHGs include CO_2_, methane (CH_4_), hydrofluorocarbons (HFCs), nitrous oxide (N_2_O), perfluorocarbons (PFCs), and sulphur hexafluoride (SF_6_) [24]. Other environmental data, such as NH_3_, wind speed, temperature, and humidity, are often packed in the same measurement data payload.

Assuming the smallest PHY payload capacity of 59 bytes with a data rate of 0 (SF12/125 kHz), a possible composition of sensor measurement is presented in Table 8 below.

Section 3.4 determines the maximum amount of GHG sensor measurements that can be inserted into a LoRaWAN packet and how frequently a LoRaWAN node can transmit this packet to the gateway. As shown, even with SF12, a payload size of 59 bytes is more than sufficient to send 9 types of unencoded 4 bytes (or 18 types of coded 2 bytes) of GHGs and other environmental data in one LoRaWAN packet with a ~260 s time interval. However, this view is valid only under the ideal condition of a single-node network, where contention and interference are absent. More realistic scenarios involving high node densities are examined in Section 4.2, Section 4.3, Section 4.4

### 3.5. DR of Sensor Measurement Update

Due to the close coupling between LoRaWAN packet uplink transmissions and sensor measurement updates in this study, the two terms will be used interchangeably hereafter. The sensor measurement update DR defines the percentage of sensor measurement updates that are successfully received by the network server from all nodes over a period T, which is represented by

(9) $${DR}^{T}=\frac{\sum_{i}^{n}N_{i}^{r}}{\sum_{i}^{n}N_{i}^{s}}$$

where $N_{i}^{s}$ is the total of number of sensor measurement updates sent by node I, $N_{i}^{r}$is the total of number of measurement updates that are successfully received by the network server, and n is the total number of nodes in the network.

### 3.6. RFEC per Node per Second

FloRa provides an energy consumer module to estimate the energy consumed by a LoRaWAN node or, more specifically, the radio module. *RFEC* is calculated based on the amount of time that the LoRa radio is in transmit, receive, and sleep states. The instantaneous power consumption P of the radio module can be represented by

(10) $$P=V\left\{\begin{array}{c} I_{rx},\;\;\;\;\;\;\;\;\;\;\;if\;s=receiving\\ I_{tx}\left(TP\right),\;\;\;if\;s=transmitting\\ {\;\;\;I}_{sleep},\;\;\;\;\;\;\;\;\;\;if\;s=sleeping \end{array}\right.$$

where V is the supply voltage, $I_{rx}$ is the current draw in receive mode, $I_{tx}\left(TP\right)$ is the current draw in the transmit state (s), which in turn depends on *TP*, and $I_{sleep}$ is the current draw during the sleep state. From (11), the total *RFEC* over a time period *T* for node *i* can be calculated as follows:

(11) $${RFEC}_{i}^{T}=\int_{0}^{T}V[I_{rx}*\delta \left(s=rx\right)+I_{tx}\left(TP\right)*\delta \left(s=tx\right)+I_{sleep}*\delta (s=sleep)]dt$$

where $\delta \left(condition\right)$ = 1 if true, and otherwise 0.

Hence, the average RF energy consumed per successful measurement update delivery can be computed by dividing the total energy used by all LoRaWAN nodes over a period *T* with the total number of sensor measurement updates received by the network server. This can be represented by

(12) $${RFEC}_{per\;update}^{T}=\sum_{i}^{n}\frac{{RFEC}_{i}^{T}}{N_{i}^{r}}$$

where *n* is the total number of nodes.

### 3.7. Adaptive Data Rate Algorithm (ADR)

With the aim of optimizing the network capacity, transmission reliability, and nodes’ energy efficiency, the LoRaWAN protocol defines a link-based ADR scheme which can dynamically control the uplink TP and SF of LoRa nodes [10]. If an end node requires the ADR service, it sets the ADR flag in the uplink message. Upon reading the flag ON, the network server can then control the end node’s transmission parameters. Therefore, with this capability, it is now possible for LoRaWAN network operators to design their own ADR algorithms. This section first investigates the implementation of the ADR algorithm into the existing LoRaWAN according to LoRa alliance [25]. Then a number of enhancements are proposed to compare and contrast their performances under different node densities, sensor update rates, and payload sizes.

#### 3.7.1. ADR-NODE and ADR-NET

The existing ADR algorithm adopted by previous studies can be separated into two parts: one operates at the node (ADR-NODE) and another is at the network server end (ADR-NET). While the ADR algorithm on the network server can adjust the spreading factor (SF) and the transmission power (TP), the node-side algorithm can only adapt the SF [10]. In this work, the ADR-NODE algorithm is maintained, and enhancements are only performed at the network server end.

As described in [10], ADR-NET first estimates the uplink budget of the sensor node by measuring the SNR of the received packets and then sends the newly calculated parameters, such as TP and SF, to the node via the downlink frame. The key parameter of the uplink budget, i.e., the SNR margin, is calculated based on the known minimum required SNR and device margin. Equation (13) defines how ${SNR}_{margin}$ is calculated:

(13) $${SNR}_{margin}={SNR}_{received}-{SNR}_{threshold}-{Margin}_{default}$$

where ${SNR}_{received}$ is the actual SNR of a packet received at the gateway, and ${SNR}_{threshold}$ is the minimum required SNR (or SNIR if interference is present) for the packet to be decoded at a certain SF. ${Margin}_{default}$ is a safety margin, taking into account the environmental variability.

#### 3.7.2. ADR-NET-Mean (ADR+)

Reference [10] proposed a modified algorithm called ADR+, where instead of using the max value for ${SNR}_{received}$, they replaced it with a mean value. This approach is more conservative for estimating ${SNR}_{margin}$ in an environment with high variability. This algorithm is also used to benchmark the rest of the algorithms designed in this paper. The pseudocode of the ADR+ algorithm is presented in Algorithm 1.
**Algorithm 1: ADR-NET-Mean (ADR+)**SNR_m_ = mean(SNR of last 20 frames)SNR_req_ = demodulation floor (current data rate)deviceMargin = 10SNR_margin_ = SNR_m_ − SNR_req_ − deviceMarginsteps = floor(SNR_margin_/3)# The remaining part is the same as ADR-NET

#### 3.7.3. ADR-NET-Linear Weighted Moving Average (LWMA)

In case of high variability, heavier weights can be assigned to the SNR of more recent frames, referred to as Linear Weighted Moving Average. As depicted in Equation (14), starting from the 20th frame before the current frame and moving up to the current frame, the weight of the SNR for each frame is incremented by one relative to the previous frame. This method’s advantage lies in its ability to more effectively capture recent changes in the signal. Unlike a simple average, which treats all data points equally, this algorithm gives more weight to more recent frames, giving a better estimate of the current signal state. This weighting approach can also reduce the impact of noise, as older frames may be more susceptible to noise interference compared to more recent frames that are closer to the signal’s true state. Note that the frame count is kept at 20 in order to be consistent with the original ADR+ algorithm. The pseudocode of the ADR-NET-LWMA algorithm is presented in Algorithm 2.

(14) $${SNR}_{m}=\frac{{SNR}_{0}\times 1+{SNR}_{1}\times 2+\cdots +{SNR}_{19}\times 20}{1+2+\cdots +20}$$

**Algorithm 2: ADR-NET-Linear Weighted Moving Average (LWMA)**totalSNR = 0weights = 0currentWeight = 1for frame[I − 19] to frame[i] totalSNR += SNR × currentWeight weights += currentWeight currentWeight += 1SNR_m_ = totalSNR/weights# The remaining part is the same as ADR+

#### 3.7.4. ADR-NET-Exponential Moving Average (EMA)

To further expand on the more recent SNR, an Exponential Moving Average method is employed. The algorithm begins by initializing the EMA to the value of the last frame’s SNR, then iterates from the 20th frame before the current frame to the current frame, using a smoothing factor k that is equal to $\frac{2}{21}$ to update the EMA, as shown in Equation (15). The final SNR (${SNR}_{m}$) is set to the last calculated EMA value, and the remaining part of the process is the same as ADR+. The pseudocode of the ADR-NET-EMA algorithm is presented in Algorithm 3.

(15) $${EMA}_{n}={SNR}_{n}\times k+{EMA}_{n-1}\times \left(1-k\right)$$

**Algorithm 3: ADR-NET-Exponential Moving Average (EMA)**ema = last framek = 2/21for frame[I − 19] to frame[i] ema = SNR × k + ema × (1 − k)SNR_m_ = ema# The remaining part is the same as ADR+

The EMA algorithm offers several advantages over simple and weighted averages, including greater responsiveness to recent data changes and a smoother output that reduces the impact of outliers and reduces lag, which might be suitable for environments with high variability and noise.

#### 3.7.5. ADR-NET-Kaufman’s Adaptive Moving Average (KAMA)

The KAMA is a sophisticated method for calculating a moving average that adapts to the volatility of the data, making it particularly useful for analysing time series data such as SNR values. The process begins with calculating the Efficiency Ratio (ER), which is the ratio of change to volatility, where change is the absolute difference between the current SNR and the SNR from 10 frames ahead, and volatility is the sum of the absolute differences of the last ten SNR changes. The next step involves determining the Smoothing Constant (SC), which is calculated based on the ER and ranges between the fastest and slowest SC values, as shown in Equation (20). Finally, the KAMA is computed using Equation (21), which incorporates the SC to adjust the influence of the current SNR on the KAMA value. The pseudocode of the ADR-NET-KAMA algorithm is presented in Algorithm 4.

(16) $$Change=\left|{SNR}_{n}-{SNR}_{n-10}\right|$$

(17) $$Volatility=\sum_{n-10}^{n}\left|SNR_{j}-SNR_{j-1}\right|$$

(18) $$ER=\frac{Change}{Volatility\;}$$

(19) $$Fastest\;SC=\frac{2}{2+1},\;\;Slowest\;SC=\frac{2}{30+1}$$

(20) $$SC=[ER\times (Fastest\;SC-Slowest\;SC)+Slowest\;SC{]}^{2}$$

(21) $$KAMA_{n}=KAMA_{n-1}+SC\times \left(SNR-KAMA_{n-1}\right)$$

**Algorithm 4: ADR-NET-Kaufman’s Adaptive Moving Average (KAMA)**Change = abs(SNR[i] − SNR[i-10])for SNR[i-10] to SNR[i] Volatility += abs(currentSNR − previousSNR)ER = Change/VolatilityfastestSC = 2/(2 + 1)slowestSC = 2/(30 + 1)SC = pow(ER × (fastestSC − slowestSC) + slowestSC, 2)KAMA = KAMA + SC × (SNR − KAMA)SNR_m_ = KAMA# The remaining part is the same as ADR+

This method offers several advantages, including the ability to adapt to changing propagation conditions by adjusting the weight of recent data points based on volatility, providing a more responsive and accurate measure of the trend compared to traditional moving averages. In addition, KAMA reduces lag and noise, making it a promising method to adapt to a highly volatile environment.

## 4. Simulation Results

The key LoRa physical layer parameters employed in the subsequent studies are summarized in Table 1, which are derived from the SEMTECH Corporation datasheet [18] and the LoRaWAN Alliance specification [17]. In FloRa, if two packets use the same channel and SF is received by a LoRa receiver, the packet with the higher received signal strength indicator (RSSI) can be decoded, provided that its signal-to-noise and interference ratio (SNIR) exceeds the threshold.

**Figure 2:**
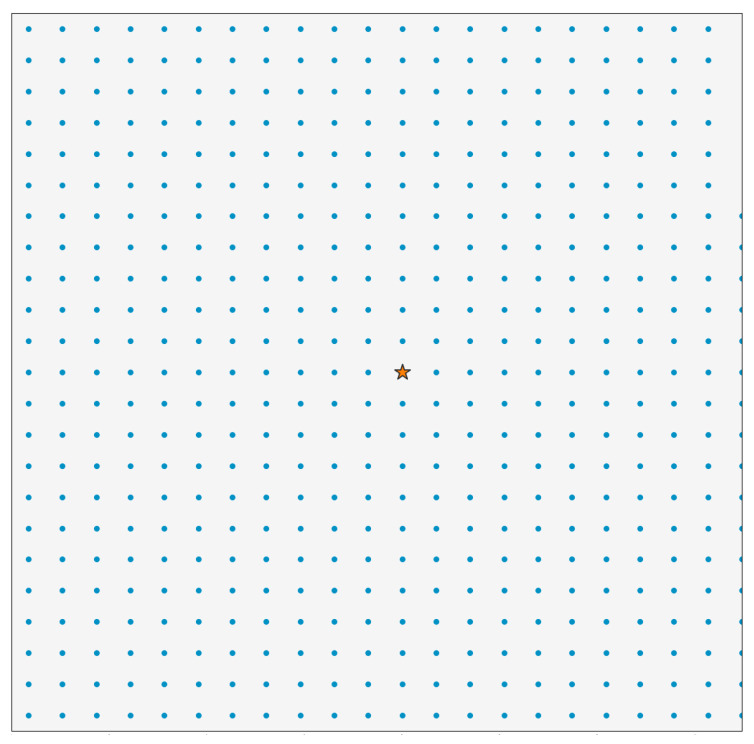
Node topology with 500 nodes uniformly distributed in 2 × 2 km^2^ area in the model.

Figure 2 illustrates the topology adopted in this paper. The network model consists of a 16 km^2^ (4 × 4 km) coverage area with a single gateway positioned at the centre. As shown in the figure, the sensor nodes are uniformly distributed throughout the area. The evaluation of a representative range of these fixed densities enabled the exploration of performance boundaries and trends that are relevant across a variety of deployment scenarios, including mixed environments.

All simulations were carried out using OMNeT++ (version 6.0.2), together with INET (version 4.4.0) and FLoRa (version 1.1.0) frameworks.

### 4.1. Verification of Single LoRaWAN Node Model

**Figure 3:**
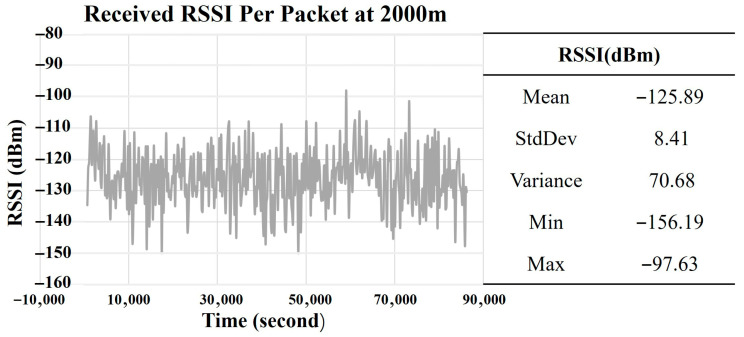
RSSI of pre-decoded packets received at 2000 m with uplink packet interval of 200 s and typical payload size of 20 bytes over period of one day with ADR+ algorithm.

Figure 3 illustrates the RSSI of pre-decoded packets received at the gateway from a node that is 2000 m away from the gateway using the ADR+ algorithm.

As shown, the received RSSI exhibits high variability, with values ranging from −97.63 to −156.19 dBm. This implies that the received RSSIs are capable of supporting transmissions from SF7 to 12 (or DR0 to DR5), which requires a minimum RSSI ranging from −121 to −137 dBm according to [18]. Table 9 presents the measured DR and RFEC over the one day period.

### 4.2. Effects of Node Densities

In this section, the total number of nodes per 4 km^2^ is increased from 100 to 500 in increments of 100 nodes. This is translated to 25, 50, 75, 100, and 125 nodes per km^2^ or average distances between nodes of ~200, 141.42, 115.47, 100, and 89.44 m, respectively. Each simulation is run over a simulation period of 3 days. Initially, all sensor nodes are set to the max TP (14 dBm) and most robust SF (SF12) according to Table 8 to ensure all nodes are within the coverage of the gateway.

**Figure 4:**
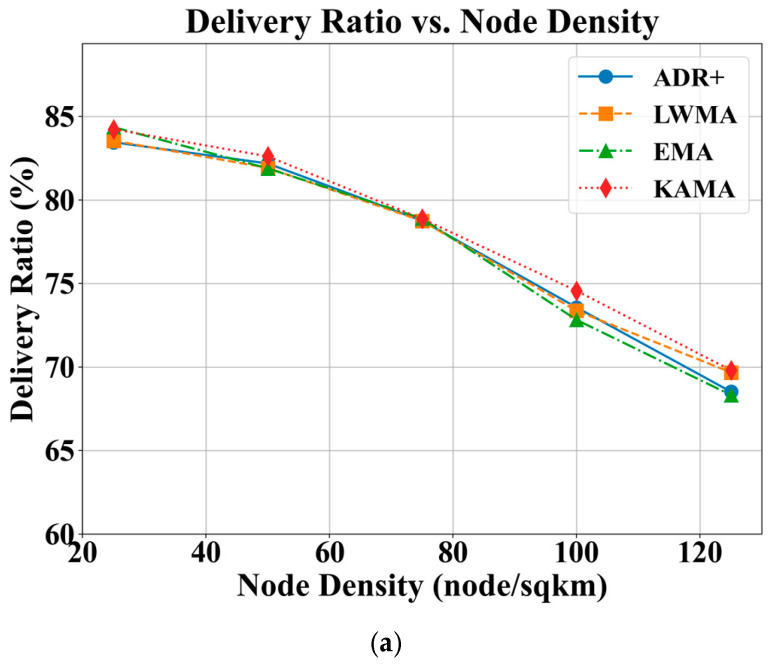

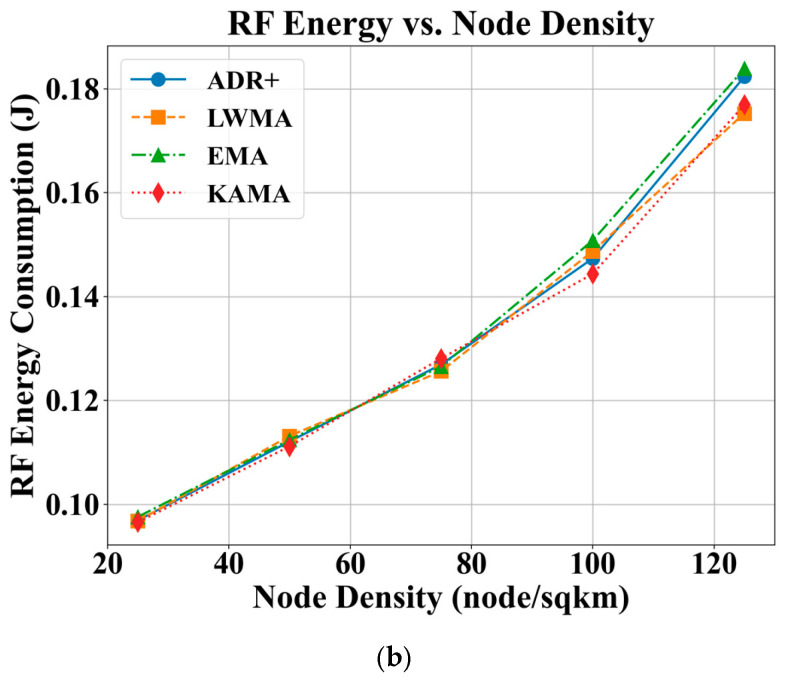
Effects of ADR-NET algorithms under varying node densities, with a sensor update interval of 600 s and a payload size of 12 bytes. (**a**) DR; (**b**) RFEC.

As depicted in Figure 4, all ADR algorithms achieve DRs exceeding 80% at lower node densities. Generally, a reduction in DR by approximately 15% is observed as the node density increases from 25 to 125, which corresponds to an average reduction of 0.15% per additional node per km^2^. The rate of DR reduction becomes more significant at higher node densities for all algorithms. The trend is expected due to higher node densities leading to increased contention and collisions, compounded by a 1% duty cycle constraint. A comparison of the algorithms reveals that KAMA, followed by LWMA, consistently provides a better DR compared to the conventional ADR+ and EMA. The observation is most notable at higher densities, where KAMA achieves a DR that is approximately 2% higher than ADR+ and EMA. Consequently, KAMA is a more suitable choice for large-scale, high-density network deployments.

An increase in RFEC is observed across all algorithms as the node density increases, with the effect becoming more pronounced at higher densities, particularly between 100 and 125 nodes per km^2^. This trend is primarily attributed to a higher rate of sensor update failures, which lead to increased energy waste due to unsuccessful transmissions. On average, the addition of 100 nodes per km^2^ results in an increase of approximately 80 mJ in RFEC per successful sensor update, which corresponds to an increase of 0.8 mJ per successful sensor update when a new node is added to the network. Among the evaluated algorithms, KAMA consistently demonstrates better energy efficiency, particularly in high-density scenarios, achieving approximately 6–7 mJ lower energy consumption per successful update compared to ADR+ and EMA.

#### SF Distribution for KAMA

**Figure 5:**
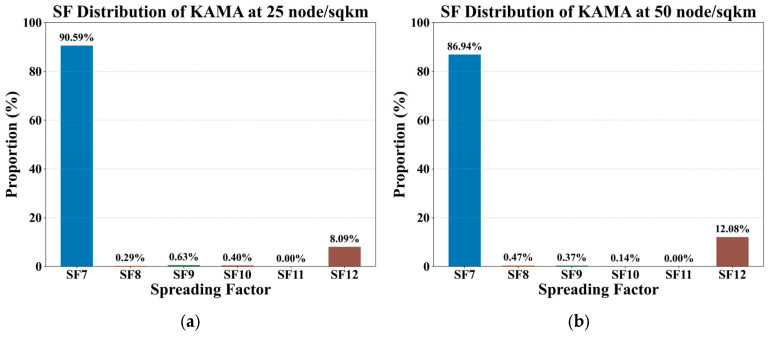

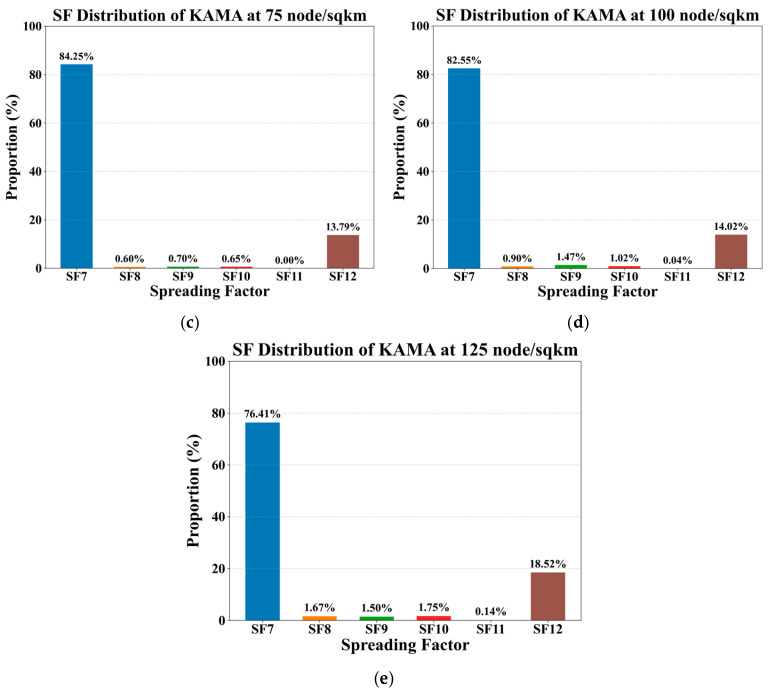
SF distribution of KAMA under varying node densities, with a sensor update interval of 600 s and a payload size of 12 bytes. (**a**) at 25 nodes/sqkm; (**b**) at 50 nodes/sqkm; (**c**) at 75 nodes/sqkm; (**d**) at 100 nodes/sqkm; (**e**) at 125 nodes/sqkm.

The SF distribution of KAMA algorithm across five node densities is illustrated in Figure 5. Across all node densities, an SF of 7 is the most utilized, followed by an SF of 12, with an SF of 11 being the least common. This is likely attributable to the relatively short distances in high-density deployments, which allows the majority of packets to successfully reach their destination using an SF of 7. As the node density increases, a corresponding decrease in the proportion of SFs with a value of 7 is observed, while the usage of other spreading factors rises. This trend suggests that the increased variation in distances between the gateway and individual nodes at higher densities requires a more diverse allocation of spreading factors to maintain robust connectivity. While this analysis only highlights KAMA, other ADR algorithms demonstrate a similar overall trend, with an SF of 7 dominating the distribution. However, KAMA exhibits a slightly higher proportion of SFs with a value of 7, which is attributed to its enhanced ability to measure and respond more accurately to SNR trends.

### 4.3. Effects of Sensor Measurement Update Rate

**Figure 6:**
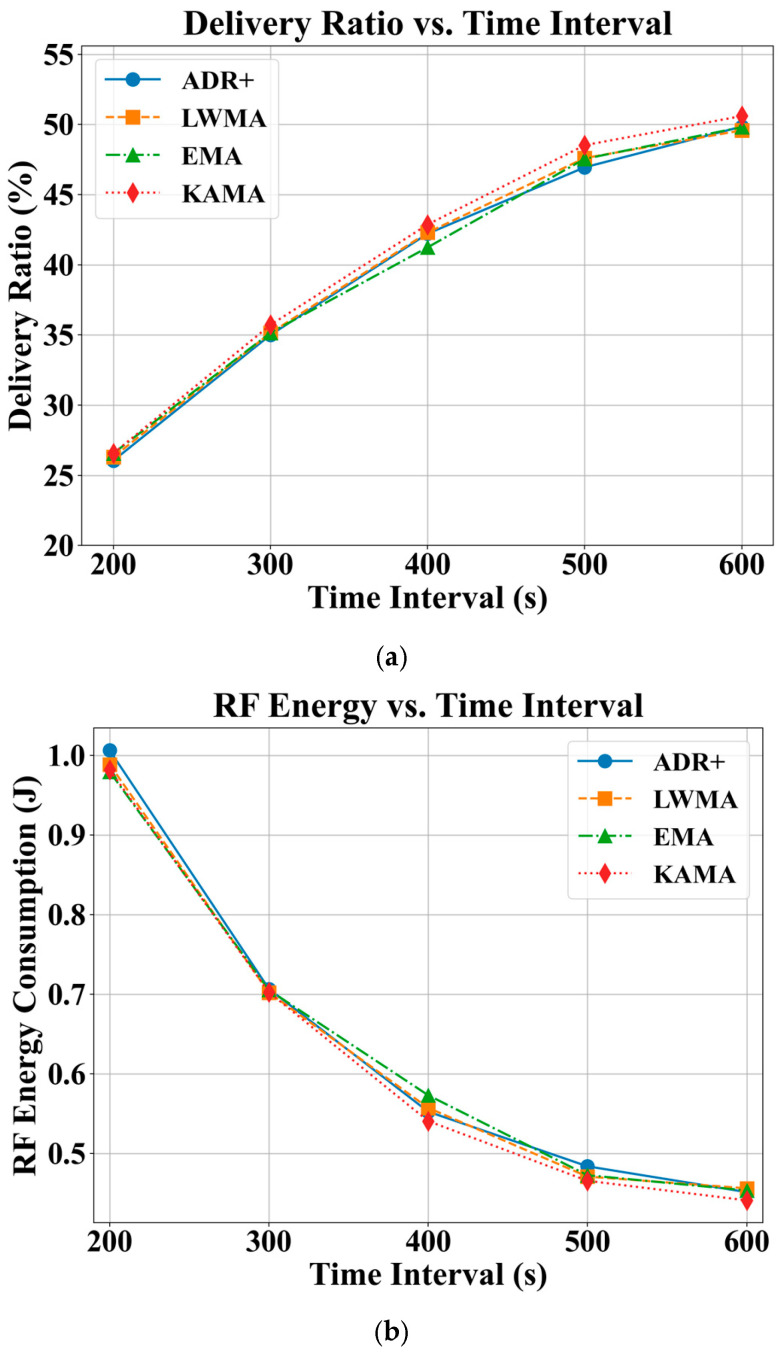
Effects of ADR-NET algorithms under varying sensor update time intervals, with payload size of 12 bytes and node density fixed at 125 nodes per km^2^ or 89.44 m average distance between nodes. (**a**) DR; (**b**) RFEC.

Extending the sensor measurement update interval from 200 s to 600 s resulted in a near-linear improvement in DR across all evaluated algorithms, as shown in Figure 6a. However, the overall DR remained below 50%, indicating that high-frequency updates in the range of a few hundred seconds are not efficient and are therefore not recommended for high-density deployments. A comparative analysis reveals that KAMA consistently achieves a 1–2% higher DR than the other algorithms. As illustrated in Figure 6b, sensor updates at a 200 s interval incur an RF energy consumption of approximately 1 J per update, which is considerably high. Notably, increasing the update interval beyond 500 s yields a more than 50% reduction in RFEC. Consistent with its DR performance, KAMA also demonstrates better energy efficiency, with 10–15 mJ lower RFEC compared to the other algorithms.

### 4.4. Effects of Sensor Measurement Payload Size

To reflect a range of practical use cases, three representative payload levels were simulated: a low load (14 bytes), a medium load (36 bytes, representing five types of unencoded 4-byte sensor measurements, as detailed in Section 3.4.2), and a maximum load (59 bytes). Although the payload was not varied dynamically to avoid additional complexity, this fixed-load approach provides valuable insights into network performance boundaries under different payload conditions.

**Figure 7:**
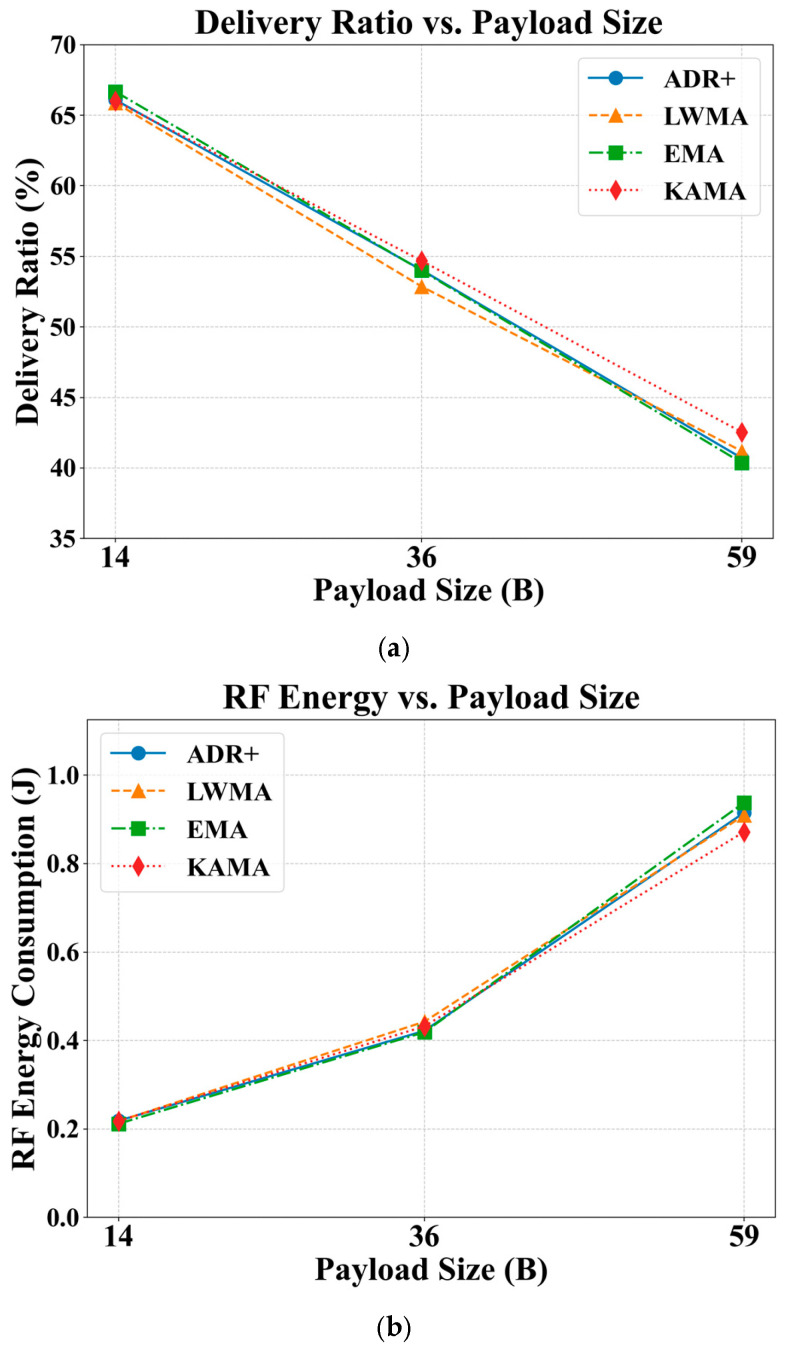
Effects of ADR-NET algorithms under varying sensor payload sizes with sensor update interval of 600 s and node density of 125 nodes per km^2^ or 89.44 m average distance between nodes: (**a**) on DR; (**b**) on RFEC.

As depicted in Figure 7, the payload size has a significant effect on both the DR and RFEC. Even with only 14 bytes of payload, the DR is only around 65%. A near-linear decrease in DR of approximately 25% is observed as the payload size increases from 14 to 59 bytes, representing a 2.24% reduction in DR of approximately 0.56% per byte. When translated to typical uncoded sensor measurements, this represents a 2.24% drop in DR when a sensor measurement is added. A comparative analysis across the algorithms shows that KAMA generally offers higher DR, with an improvement of up to 2%.

Figure 7b shows that the RFEC increases with payload size, with this effect becoming more pronounced at higher payloads. Specifically, a consumption of approximately 10 mJ per byte is observed when the payload increases from 14 to 36 bytes, while this rises to approximately 20 mJ per byte for the increase from 36 to 59 bytes. Comparatively, KAMA consistently offers a better RFEC, achieving a reduction in energy consumption of between 40 and 70 mJ as the payload increases from 14 to 59 bytes. Although the findings generally indicate that the payload size has significant adverse effects on both the DR and RFEC, they also demonstrate that KAMA remains a promising algorithm across a wide range of payload conditions.

## 5. Conclusions and Future Work

This paper has examined the key requirements for effective continuous GHG monitoring from the perspective of LoRaWAN communication technology. A comprehensive evaluation of LoRaWAN’s sensor measurement update capacity was conducted, focusing on the influence of varying sensor update rates and data payload sizes. A simulation-based study was carried out to assess the effects of the node density, update frequency, and payload size on two critical performance metrics: DR and RFEC per successful sensor update. The findings indicate that all three parameters significantly affect both the reliability and energy efficiency. In particular, high-frequency updates in the order of several hundred seconds lead to substantial degradation in DR and are therefore not recommended for high-density deployments. Among the evaluated adaptive data rate strategies, the ADR-NODE-KAMA algorithm consistently demonstrated better performance across a wide range of operating conditions, offering improved delivery reliability and reduced energy consumption.

To better reflect real-world deployment scenarios, future work should investigate non-uniform or adaptive-node-density configurations that capture the spatial variability that is commonly observed in urban environments. From a sensor deployment logistics perspective, mixed-use settings, such as industrial and residential zones, can significantly influence node placement strategies and affect the spatial distribution of sensing nodes, particularly in large-scale urban or semi-rural GHG monitoring applications. Network maintenance costs also need to be considered, as they are influenced by routine requirements such as battery replacement and node/gateway maintenance, including firmware updates, all of which become increasingly significant as the node density grows. Moreover, continuous and high-temporal-resolution GHG monitoring generates substantial volumes of data and may require edge preprocessing and scalable cloud-based analytics pipelines to support decision-making. These highlight the need for future work to incorporate system-level cost modelling and data management frameworks to ensure end-to-end deployment viability.

In addition, the current simulation framework should be extended to include multi-gateway deployments, enabling the investigation of inter-gateway coordination, overlapping coverage, and gateway density on network scalability and reliability. Moreover, hybrid ADR schemes that combine the strengths of multiple ADR strategies could be developed to adapt more flexibly to varying network conditions. Advanced machine learning-based techniques such as XGBoost, Naïve Bayes, and k-NN [26] can be used to optimize TP and/or SF via classification. Time series models such as LSTM can be used to learn temporal patterns, i.e., predicting sensor measurements in order to decide whether to transmit.

In terms of energy costs, future work should incorporate a more comprehensive node-level energy model that accounts for the effects of sampling intervals, sensor preheating, and measurement energy across different sensor subsystems, such as electrochemical and optical gas sensors. To improve statistical rigour, significance tests such as *t*-tests or ANOVA should also be employed to further validate the performance differences among ADR algorithms.

## Figures and Tables

**Table 1:** Parameters of LoRa physical layer (uplink).

Parameter	Value	Description
Carrier Frequency	490 MHz	CN470–510 MHz band
Channel Bandwidth	125 kHz	Starts at 470.3 MHz and increases linearly by 200 kHz to 489.3 MHz [17] ^1^
Coding Rate	4/5	Typical
Spreading Factor	Varies between 7 and 12	Controlled by ADR in this study
Transmit Power	Varies between 2 and 14 dBm	
Duty Cycle	1%	General condition; 10% is allowed in 779–787 MHz band
Receiver Sensitivity at Gateway	−137 dBm	Semtech RFS_L123_LF [18]
SNIR Threshold	−7.5 dB	For SF7 [17]
Energy Detection	−110 dBm	For interference avoidance
Capture Effect Margin	6 dB	If overlaps ≥5 preamble symbols, and received power is at least 6 dBm greater [10]

^1^ Channel Indexes of 6 to 38 and 45 to 77 are mainly used by China Electric Power. Hence, in the areas where these channels are used by China Electric Power, they should be disabled

**Table 2:** Parameter settings of the propagation model for an urban scenario.

Parameter	Value	Description
n	3.5	Path loss exponent assuming moderately dense urban areas which typically range between 2 and 4 [14]
$\sigma \left(dB\right)$	8	Standard deviation of shadow fading (log-normal fading) indicating variability in path loss due to urban clutter
$d_{0}\left(m\right)$	1000	Reference distance
$\bar{PL}\left(d_{0}\right)\left(dB\right)$	119.5	$\mathrm{Path}\;\mathrm{loss}\;\mathrm{at}\;d_{0}$
$h_{gw}$(m)	30	Antenna height at gateway assuming a typical rooftop-mount
$h_{node}$(m)	1.5	Antenna height at typical ground-level sensor

**Table 3:** Time on air (ToA).

SF	$T_{s}$ (ms)	DE	ToA (ms)
7	1.024	0	107.776
8	2.048	0	195.072
9	4.096	0	349.184
10	8.192	0	657.408
11	16.384	1	1396.736
12	32.768	1	2629.632

A 59 B payload is the maximum payload size in the CN470–510 band.

**Table 4:** Minimum waiting time per SF under 1% duty cycle.

SF	$T_{wait}$(s)
7	10.67
8	19.312
9	34.569
10	65.083
11	138.277
12	260.334

**Table 5:** Uplink packet structure and field sizes.

Element	Typical Size (Bytes)
PHY Header and CRC	2 to 4
**PHY Payload**	
MAC Header (MHDR)	2
**MAC Payload**	
Device Address (DevAddr)	4
Frame Control (FCtrl)	1
Frame Counter (FCnt)	2
Frame Optional (Fopts)	0 to 15
Frame Port (Fport)	1
Frame Payload	Varies according to data rate
MIC	4

**Table 6:** Maximum payload size in CN470–510 band [11].

$\mathrm{Data}\;\mathrm{Rate}\;(R_{b}$ ^1^)	M (Bytes)	N (Bytes)	Configuration
0	59	51	SF12
1	59	51	SF11
2	59	51	SF10
3	123	115	SF9
4	230	222	SF8
5	230	222	SF7

^1^ $R_{b}=SF*\frac{\left[\frac{4}{4+CR}\right]}{\frac{2^{SF}}{BW}}$.

**Table 7:** Typical measurement data size for GHGs and associated environmental data [25].

Gas Type/Other Environmental Data	Detection Unit	Encoding Type	Data Size (Bytes)
CO_2_	ppm	Can be uint16, int16, float16, or float32 depending on required range and precision	2~4 bytes
CH_4_	ppm		
N_2_O	mg/m^3^ (varies)		
HFCs	ppt or ng/m^3^		
PFCs			
NF_3_			
SF_6_	ppt or ppb		
Water Vapour	ng/m^3^		
Wind	m/s		
Humidity	%		
Temperature	°C		
Pressure	mb, hPa or Pa		
Precipitation	mm		
Solar Radiation	W/m^2^		

**Table 8:** Typical sensor measurement composition in LoRaWAN packet.

Field	Size (Bytes)	Description
MAC, Frame Header, and MIC	14	Assuming no optional frame control
**MAC Frame Payload**		
CO_2_	4	Assuming single-precision float (float32)
CH_4_	4	Assuming single-precision float (float32)
HFCs/PFCs	4	Assuming single-precision float (float32)
Flags	1	Status flags (e.g., sensor errors, battery)
Timestamp	4	Unix timestamp (optional)
Location	4	GPS latitude and longitude
**Total**	**35**	**14 + 21**
Payload Capacity	59	Maximum possible for SF12
Remaining	24	At least 6 types of sensor measurements

**Table 9:** Performance metrics per node (simulation).

Metric	Value	Remarks
DR	0.42 (42%)	Only 362 out of 861 are decoded successfully due to high variance
RFEC	75.20 mJ	Measured in millijoules, based on a supply voltage of 3.3 V and the current draw profile from [18]

## Data Availability

The data presented in this study are available on request from the corresponding author.

## Abbreviations

The following abbreviations are used in this manuscript:

GHG	Greenhouse gas
CTMs	Chemical transport models
DR	Delivery ratio
RFEC	RF energy consumption
ADR	Adaptive data rate
VNS	Variable neighbourhood search
SF	Spreading factor
DER	Data extraction rate
NEC	Network energy consumption
FLoRa	Framework of LoRa
TP	Transmit power
MHDR	MAC header
FHDR	Frame header
DevAddr	Device address
FCtrl	Frame control
Fopts	Frame optional
RSSI	Received signal strength indicator
SNIR	Signal-to-noise and interference ratio
